# Near-peer teaching in problem-based learning: Perspectives from tutors and tutees

**DOI:** 10.1371/journal.pone.0278256

**Published:** 2022-12-14

**Authors:** Binbin Zheng, Zilu Wang

**Affiliations:** Bau Institute of Medical and Health Sciences Education, Li Ka Shing Faculty of Medicine, The University of Hong Kong, Hong Kong, Hong Kong; Ege University Faculty of Medicine, TURKEY

## Abstract

**Introduction:**

This study explores how tutors and tutees perceived their teaching and learning experience in a near-peer teaching programme within a formal undergraduate medical-education curriculum.

**Methods:**

This mixed-methods study was conducted in an Asian medical school. First, a survey was administered to two groups of students, one that had been tutored by near-peers, and another with faculty tutors. Then, the near-peer tutors were interviewed and wrote reflection essays that the researchers collected. Quantitative analysis was used to analyse the survey responses, and qualitative analysis to analyse the interview and reflection data.

**Results:**

Our study found no difference between near-peer tutees’ and faculty tutees’ perceptions of either tutor facilitation or tutor behaviours. Also, when near-peer tutors explained how their experience of delivering tutoring had influenced their professional-identity formation, they highlighted that they had gained skills important to their future careers as medical educators.

**Conclusion:**

Integrating near-peer teaching into undergraduate medical curricula could be beneficial to both tutors and tutees because of the social, cognitive, and professional congruence between these two groups, and due to its likely positive influence on their professional-identity formation.

## Introduction

Near-peer teaching (NPT), “in which one student teaches one or more fellow students” [1], has been adopted in many medical schools to complement limited faculty-teaching resources and/or to help medical students achieve teaching competency and prepare them for lifelong teaching [2]. Though tutors and tutees can be on exactly the same educational level, they are more usually “near peers”: i.e., the tutor is at a slightly more advanced stage of training [1,3]. NPT can potentially benefit tutors in various ways, such as improving their content knowledge, communication skills, and/or teaching motivation [4–8]. Also, as Joubert (1899) noted, “to teach is to learn twice” (see also [9]). That is, before teaching, near-peer tutors need to gain a comprehensive understanding of the content knowledge being taught, and anticipate the questions tutees might ask [10]; and during teaching, such tutors must be able to verbalise information in a clear and easy-to-understand way, assess their tutees’ learning progress, and provide effective feedback. Thus, the entire NPT process can help tutors deepen their own knowledge, recognise their shortcomings, and reflect on their own learning [2,4,10]. Moreover, as near-peer tutors need to think metacognitively about how to help others learn, NPT could further improve their self-regulated metacognitive learning skills such as planning, monitoring, and evaluating, as well as their cognitive ones such as reciting, elaborating, organising, applying, and inferring [1].

As a near-peer tutor, a student automatically positions him- or herself in a teaching role. Role theory holds that, when placed in a position of authority, a person tends to gain self-confidence and self-esteem [6], as well as motivation to retain that position [11]. This role-shifting further encourages those delivering NPT to be well prepared. In addition, the valuable teaching and communication skills they gain via NPT–such as clearly conveying information, conducting assessments, and providing feedback–are, over the long term, important for them to have as medical educators and physicians [12,13]. Unsurprisingly, research has suggested that NPT positively influences students’ desire and motivation to engage in future teaching activities [2].

Additionally, training senior medical-school students to become tutors to other undergraduate medical students could help meet the increasing demand for doctors in Hong Kong and the associated needs of its growing medical-student population [14]. Because of the student-centredness of problem-based learning (PBL), which is widely adopted in medical school curricular, it has been argued that the social congruence of tutors–i.e., their interpersonal qualities such as the ability to communicate empathetically with students–profoundly influences student learning [15,16]. Moreover, such influence may be even greater than that of tutors’ content knowledge and facilitating skills, due to the importance of creating an unthreatening environment marked by student engagement and the open exchange of ideas [15,17].

Nevertheless, little has hitherto been written about the training tutors receive prior to delivering peer-tutoring programmes in medical education. The present study helps to fill that gap through 1) the development of an NPT programme that involves systematic training, thoughtful preparation, and ongoing support for near-peer tutors; 2) the careful implementation of a PBL curriculum; and 3) a thorough evaluation of the programme’s effectiveness. This project also aligns well with the concept of “students as partners” [18]: i.e., that involving students in their own curricula is likely to increase the accountability of their curricular decisions, enhance their engagement and responsibility for learning, and strengthen their professional-identity formation [19]. Through analysing the implementation and outcomes of our NPT programme, we hope to learn how NPT experiences could enhance medical students’ professional-identity development as well as their learning. And, from an institutional perspective, this study should also yield insights into how to reform and improve NPT practices.

Four research questions were explored in this study:

1. Did PBL students have different perceptions of staff tutors’ vs. near-peer tutors’ a) facilitation and b) skills?
2. How did near-peer tutors perceive the influence of the NPT experience on their professional-identity development?
3. What strengths did near-peer tutors perceive themselves to have, as compared to faculty tutors?
4. What institutional and faculty support did near-peer tutors consider important?

## Methods

### Context

This study was conducted in a Hong Kong medical school where, in the 2021–22 academic year, small-group PBL tutorials were delivered to all 297 first-year students in the Bachelor of Medicine and Bachelor of Surgery (MBBS) programme. For this purpose, students were randomly assigned into 28 groups, each with 10 or 11 members. Each PBL case included three two-hour tutorials over a two-week period, in which each group’s members and their tutor discussed the entire process of listening to patient complaints, history-taking, test-results presentation, differential diagnosis, and creating a management plan. The tutors included clinical educators, basic-science educators, and occasionally, social-science educators. This study’s focal case, on sleep apnoea, was the first one in the second semester and part of the course’s Cardiopulmonary and Renal Systems block.

### Procedure

Our recruitment of near-peer tutors started three months prior to the launch of the targeted PBL tutorials. In October 2021, an email invitation was delivered to all of the medical school’s Year 3–6 MBBS students, of whom 15 signed up. A peer-teaching certificate programme was provided in November and December 2021 as a condition of their eligibility to become near-peer tutors. The certificate programme consisted of four 90-minute sessions led by two experienced teacher-educators, covering 1) how students learn, 2) supporting student learning, 3) facilitating strategies for small group discussion, and 4) PBL simulations. The purpose of this was to equip students with the key teaching and facilitating skills required to conduct PBL facilitation. Because four of the original 15 volunteers failed to participate in the certificate programme, a total of 11 near-peer tutors were available for our study. These 11 were asked to submit a reflective essay as the certificate programme’s final assignment. Among the 11 near-peer tutor participants, eight were male and three, female. Eight were clinical students in their fourth (N = 3) and fifth (N = 5) years of study, while the other three were third-year students engaged in intercalation, research internship, or service work. Nine were participating in an NPT programme for the first time.

Before the NPT programme commenced, we familarised the near-peer tutors with the ground rules they should introduce at the beginning of their tutoring, gave them a basic rundown of each tutorial, and provided them with a tutor guide including some suggested guiding questions they could ask during PBL tutorials.

After each of the first and second tutorials, we held debriefing sessions in which the near-peer tutors shared teaching tips and tactics, the challenges they faced, and how they planned to overcome those challenges in their next tutorials.

### Data collection

This study was approved by the target university’s Human Research Ethics Committee (Project Reference Number: EA210511). We included three data sources in it for the purpose of data triangulation.

#### Student survey

At the end of the third tutorial, an online survey was sent out to all 297 first-year MBBS students. Among them, 212 students–i.e., 76 from groups with near-peer tutors, and 136 from groups with staff tutors–provided valid responses, representing a response rate of 73%. The survey’s 24 items, all of which were related to student perceptions of tutors, were adopted from previous literature [15,16] and answered using a five-point Likert scale ranging from 1 = strongly disagree to 5 = strongly agree. The three dimensions covered by the items were *facilitation* (e.g., “The tutor stimulated us to understand underlying mechanisms or theories”); *cognitive congruence* (e.g., “The tutor used his/her subject-matter knowledge to guide the group”); and *social congruence* (e.g., “The tutor took time to answer questions”). Two open-ended questions regarding the advantages and drawbacks of having near-peer tutors were also asked, but only to the cohort that received NPT.

#### Near-peer tutor interviews

One week after the third tutorial, we invited each of our 11 near-peer tutor participants to an anonymous one-on-one semi-structured interview with the first author, lasting approximately 30 minutes. Seven of them agreed to participate and signed the consent form. The interviews focused on near-peer tutors’ motivation to participate in the programme; their overall teaching experience; and how they thought such experience had influenced or might influence their teaching, learning, and future career prospects. All interviews were audiotaped and transcribed verbatim with the participants’ permission.

#### Near-peer tutors’ reflective essays

Of the 11 tutor participants, 10 submitted reflective essays as their final assignment in the above-mentioned peer-teaching certificate programme. To stimulate their reflections, the assignment instructions contained several prompts, including but not limited to “Have you found anything you did particularly well in those teaching sessions? What did you do? Why did it work?”, and “Have you encountered any difficulties in your facilitation? How did you handle it? How would you handle it differently in the future? Why did it not work?”

### Data analysis

To answer our first research question, pre-validated survey items on tutors’ facilitation skills, social congruence, and cognitive congruence were compared using independent-samples *t*-testing.

To answer our second, third, and fourth research questions, thematic analysis [20] conducted in an inductive manner was applied to both our types of qualitative data. Thematic analysis can help understand the impacts of a programme from the perspective of those experiencing it [21], while also providing insights into whether the processes of analysis are credible [22]. This approach had been widely adopted as a qualitative methodology in investigations of the effectiveness and outcomes of health-professions education programmes [23].

**Fig 1:**
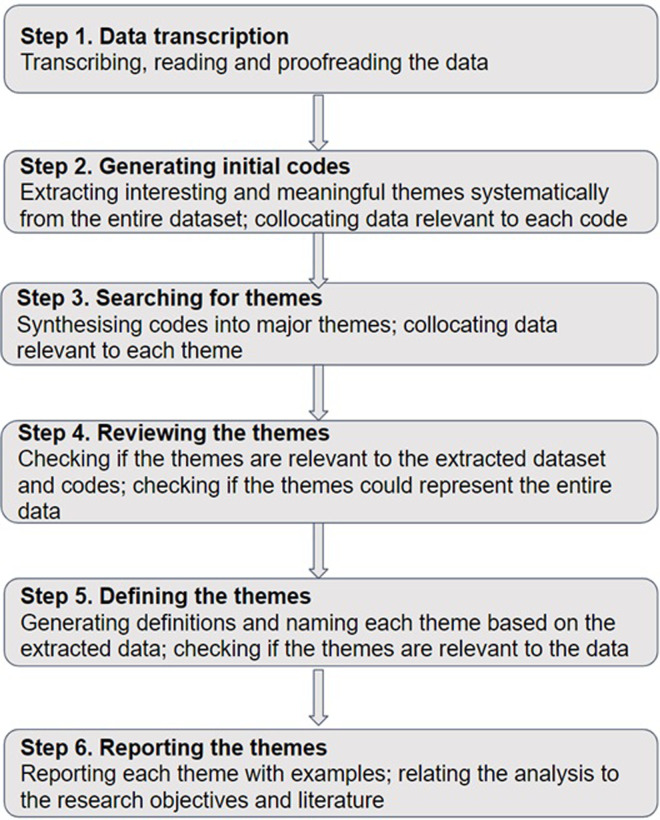
Processes of thematic analysis [24,25].

Specifically, the present study followed the process of thematic analysis recommended by Braun and Clarke [24] and summarised in Fig 1. In this process, initial thematic interpretation was performed by a trained educational researcher who was not involved in developing the interview protocol (ZW), and the codes were further cross-validated by the first author (BZ) until consensus was reached. Both these researchers are from social-science backgrounds and did not have any conflicts of interests or teaching interactions with the near-peer tutors, other than the training certificate programme. Thus, the researchers took an objective observer’s perspective when interviewing and when analysing the data.

## Results

### Students’ perceptions of near-peer tutors’ and faculty tutors’ facilitation

The results of independent-samples *t*-testing conducted to compare students’ perceptions of staff tutors’ and near-peer tutors’ facilitating skills are shown in Table 1.

**Table 1:** Student perceptions of staff tutors’ vs. near-peer tutors’ facilitation and skills.

	Students from groups with staff tutors		Students from groups with near-peer tutors			
	Mean	*SD*	Mean	*SD*	*t-*test	*p*
**Tutor-level factors**	4.25	.60	4.16	.59	1.03	.306
**Tutor facilitation**	4.24	.62	4.13	.61	1.27	.205
**Tutor behaviour**	4.25	.59	4.17	.59	.93	.355
Cognitive congruence	4.23	.62	4.20	.59	.69	.715
Social congruence	4.26	.60	4.16	.61	1.20	.230
**Outcome**	4.23	.61	4.17	.60	.66	.512
***N***	136		76			

*Note*. *p < .05, **p < .01,***p < .001; SD = standard deviation.

The results indicated that there was no significant difference in the staff-tutored and near-peer-tutored cohorts’ ratings of tutor-level factors (staff: *M* = 4.25, *SD* = .60; near-peers: *M* = 4.16, *SD* = .59; *t*(210) = 1.03, *p* = .306. The two cohorts also did not differ significantly in their perceptions of tutor facilitation (*t*(210) = 1.27, *p* = .205), tutor behaviour (*t*(210) = 0.93, *p* = .355), or the overall PBL experience (*t*(210) = 0.66, *p* = .512).

While no differences in perceptions of near-peer tutors and faculty tutors were detected from the student surveys’ Likert-scaled items, some students did mention in their open-ended survey questions that they felt near-peer tutors understood their “needs and struggles” better, and were generally more “approachable”, which helped create a “less stressful” and “more relaxing” environment. Some also mentioned that near-peer tutors shared effective study tactics whose relevance transcended the PBL curriculum.

### Near-peer tutors’ perceptions

We summarised the near-peer tutors’ interview data and reflective-essay data into three themes with a total of nine sub-themes, which are presented in depth below.

### Preparation for future careers

#### Teaching is part of being a doctor

Almost all of the interviewees mentioned preparing for their future careers as one of their strongest motivations for proactive participation in the NPT programme.

*I think* ***teaching is an important part of becoming a doctor*.** *Firstly*, *it is about* ***responsibility*.** *Because medicine is about practice and experience*, *it is a very important process if we are to* ***deliver skills and our own experience to future doctors***.

One near-peer tutor said that initially, she had supposed that “as long as you are not working in the university hospital, you do not need to participate in any teaching stuff”. But during her clinical exposure in the hospital, she “noticed that every doctor would still have some moments in their career that require them to teach”, and that this teaching experience “directly helps train future clinicians, which is directly related to the quality of healthcare”.

#### Important skills for future careers

Most near-peer tutors also noted that their NPT experience helped them gain important skills, in addition to teaching *per se*, that would prepare them for their future careers.

*[C]ommunication is one thing that I learned in this programme*. *[… A]t the start of the tutorial*, *my instruction was not really that clear […]*. *But then*, *I also had to preserve the logistics behind the PBL*, ***it is not the tutor’s job to lead the discussion***.

*In PBLs it is* ***more like a facilitator role than direct teaching mode***. *[…] Previously*, *I tried to be more informative and tried to do more direct teaching*, *but somehow after the training I realised that it is necessary for me to assess whether they really get the point*, *and to assess their understanding of topics*. *And also […] to try to accept silence*, *it is the most important topic*.

Some also mentioned having learned “a more systematic way to teach”, in which every time students state their views or opinions, the tutor encourages them to “quote references […] and try to illustrate why this point is valid, and then explain and interpret the […] data”. Some near-peer tutors also mentioned learning “how to ask questions that can stimulate student thinking”, “pay attention to listeners’ needs”, “ask more ‘why’ questions”, and encourage students to “listen to other students’ points and build on them”.

Eventually, some said, these skills gained from NPT could help them provide better healthcare. One further suggested that their NPT experience had made them even more determined to become a doctor in a teaching hospital:

*The doctors in [Name of local teaching hospital] fulfil three roles*: ***clinician*, *teacher and scientist***. *Although the work hours might be longer and the environment harsher*, *this programme further confirmed me in my dream of working in a teaching hospital[*.*]*

#### Identity shift from student to teacher

Some near-peer tutors also said that their NPT experience helped them appreciate teachers’ perspectives better, and shifted them towards that mindset. This identity shift, in turn, prompted them to revisit their experience as learners, and thus to gain a better understanding of the purpose of PBL.

*As a student*, *I was always worried about whether my answers were right or wrong*. *However*, *as a tutor*, *I find myself* ***appreciating it when students participate***, *regardless of whether their answers are right or wrong*.

*I’ve got the most out of […] the experience of being a PBL tutor and from this perspective* ***understand how different it is in terms of the PBL experience from the tutor’s*** *point of view versus when I was a participant in the pre-clinical years*.

*This positive teaching experience allows me to be more confident about engaging in education in the future*.

We also noticed that students with previous NPT experience in other teaching contexts were more adaptive to the above-mentioned identity shift.

*I had been involved in various teaching events before studying MBBS such as study groups with peers*, *knowledge-sharing with juniors*, *conducting tutorials with secondary students*, *and teaching students as a hospital staff member during their clinical attachment or placement*. *From all these experiences*, *I realised that I enjoy teaching and it* ***helps me consolidate my knowledge***, *as well as* ***facilitating my reflection on my learning experience***.

First-time near-peer tutors, in contrast, encountered more difficulties with this identity shift, expressing more stress and worry about their inability to meet students’ expectations of a tutor.

*I also suffered from* ***a worry about students’ hidden scepticism about me since I am not an experienced content expert****[*.*]*

*I also somehow felt some stress because* ***I’m still a student*** *and I will have some worries if my younger colleagues ask me some really difficult question that I maybe have some difficulty coping with […] I may lose their trust if I give them a wrong answer*, *or* ***they may feel unsatisfied if I cannot provide a good answer***.

### Near-peer tutoring’s advantages over staff tutors

#### Cognitive congruence

Some near-peer tutors mentioned that they could understand their tutees’ difficulties because they had “experienced it a few years ago”. More specifically, they could tell their students “if information is validated, concise, or relevant, what animations they can use, and how to approach PBL”. As one explained:

*When I was in Year 1*, *I struggled to understand the definitions of mixed sleep apnoea versus mixed pattern of sleep apnoea*, *and I queried the mechanisms of how OSA would lead to nocturia*. *My students shared these problems*, *so I was able to guide them through these difficult aspects of the PBL case*. *I also found the polysomnography tracings rather overwhelming when I was in Year 1*, *but through the years I have developed the skills to systematically interpret clinical investigations*. ***I explained to the students that whenever they are asked to interpret graphs or charts*, *they should follow a clear framework so that they appear more organised and will not miss important findings*** *[…]*. *Hopefully these tips will help my students with their future PBL cases and clinical scenarios*.

#### Social congruence

Most near-peer tutors also mentioned feeling that they had created an open and less stressful atmosphere, which allowed their tutees to ask questions more freely.

*One of the advantages of having NPTs rather than academically high-achieving tutors is that* ***it will be easier for my younger colleagues to ask questions*** *[… O]ne of the downsides of being a person with a very strong academic background and being very famous [… is that it makes one’s students] feel nervous while doing PBL sessions*. *If we encounter a near-peer tutor with a small age gap*, *perhaps we can have a less stressful learning experience*.

One near-peer tutor described this phenomenon as a “group identity”:

*Because we’re all students and* ***our student life is similar*** *[… tutees] may be more willing to express their ideas and participate more in the tutorial*, *in a better manner*.

Two near-peer tutors also mentioned their accessibility and relatively small age gap compared with the staff tutors, and how these factors enhanced bonding between junior and senior students:

*I also had students who messaged me after the lessons to ask for advice for medical school*, *so I think* ***having near-peer tutors is really helpful for students to get to know seniors they can turn to for advice***.

*[Tutees] may also find it* ***easier to share some non PBL related difficulties*** *and struggles to me* ***because I am also a medical student*.** *For example*, *sometimes they have one or two questions for me about the enrichment-year choices*.

#### Clinical experience

Unsurprisingly, given that eight of the 11 near-peer tutors were in their clinical years at the time of data collection, many highlighted their ability to bring in clinically relevant information, especially as compared to the basic-science faculty who made up around half of the PBL staff tutors.

*In the preclinical years*, *usually we just follow the tutor’s guidance in the discussions*, *whereas in the clinical years*, ***we can have wide-ranging discussions about different diseases around the case***. *So I think as a student peer tutor*, *we can do both*, *like we can provide some information according to the tutor guide*, *to allow a small standardised discussion among all groups*, *as well as feed in some really clinically relevant information so that the students can learn more from a case*, *rather than just prescribing content*. *I think this is the main advantage*.

*I think for NPTs*, *especially NPTs in clinical years*, *their approaches are much more clinical compared to some […] because back in the time when I did PBL*, *especially first year*, *there were quite a lot of non-clinical academics working as PBL tutors*, *so when it gets to the biology and pathophysiology of the disease*, *maybe it’s more in depth by those tutors*. *But in terms of clinical approach in a case*, *they’re not that clinical*. *You get what I mean*. *[…] I had some clinical tutors during my preclinical PBLs*. *So*, *I can tell their approach is very different*. *[…F]rom the meetings we had*, ***I think most of the NPTs kind of lean towards the clinical side*.** *Maybe not certainly better in that respect*. *So*, *when it is a case*, *which is like this one*, ***it is a playback on a consultation*.** *Maybe it is better for NPTs to lead the PBL*, *compared to non-clinical tutors*.

### Institutional and faculty support/involvement

#### Role modelling

In terms of the teaching styles they thought they had, and what kinds of teachers they wanted to be, several near-peer tutors mentioned the power of role models and how their own teachers had influenced them. For instance:

***My perfect way to teach is to teach like a peer*.** *You obviously know Drs [Name] and [Name]*. *They are so friendly that you don’t really feel their power so much*. *This kind of teaching obviously will decrease some of the power of the tutor*. *I do not want them [students] to learn because of my power*, *but because they recognise the importance of learning*. *[… ] It is* ***better to generate such a purpose of learning*, *rather than just doing what you are told***.

*When I was in Year 2*, *a professor told me*, *“The word ‘doctor’ was borrowed from Latin*, *and originally meant ‘to teach’*. *That’s what a doctor does every day*, *too”*. *What she said made me reflect on the fact that we doctors share our knowledge with our patients and even our juniors every day*. *Simply giving out pills does not make us good doctors*, *but educating our patients does*. *That being the case*, *the skills of delivering knowledge and understanding listeners’ difficulties in absorbing knowledge are both crucial*.

#### Autonomy and responsibilities

Before we launched this NPT programme, there was a discussion among the administrative team about whether faculty tutors should oversee and monitor near-peer tutors in the classroom, as a means of quality control. However, we decided to give near-peer tutors full autonomy in the classroom, and after their NPT experience, asked them how they perceived the autonomy and responsibilities that had been given to them. All expressed their appreciation of the high level of responsibility they had been accorded, and its importance to their role-building as tutors. Conversely, some said that having a staff tutor in the classroom with them would have been counterproductive. For example:

*You cannot really do a good job when you’re facing such great pressure*, *and you’re facing two types of pressure*. ***Students have some expectations of you*, *and you have some expectations of yourself*, *but the formal teacher may also have some expectations of us*.** *We will be afraid of making mistakes[*.*]*

#### Training

The peer-teaching certificate programme offered to the near-peer tutors appears to have had positive impacts on their teaching experience. They reported that the learning theories, facilitating skills and effective learning platforms introduced in that training had made them more confident and better prepared while teaching. The PBL simulations, meanwhile, encouraged their metacognitive understanding of the teaching skills they were acquiring. Further, the training helped them to shape their future roles as educators.

*I think the training […] really provided us with some knowledge and skills that are useful in PBL tutorials*.

*Before training*, *I didn’t note that the use of slides with blanks*, ***online interactive activities*** *and open-ended questions are important to facilitating students’ learning*. *Therefore*, *I tried to incorporate some of those elements*. *For example*, *I have* ***used the Poll Everywhere platform to conduct interactive activities with some test questions*** *for the participants to answer*. *This helped me to assess whether my teaching was effective and how much knowledge they had digested and absorbed*. *Also*, *during my explanations*, *I could encourage more thinking and try to identify tutees’ areas of weakness so that I could explain more about the parts they felt confused by*. *More importantly*, *it made the sessions less boring and held their attention [*…*]*. *After each session*, *I feel more comfortable[*.*]*

*Building on the skills I learned in the training sessions*, *I have practised asking questions that could stimulate students’ thinking*, *which require critical-thinking skills and clinical reasoning*. *Overall*, *the NPT teaching is enjoyable*, *since I could try something that I had never participated in before*, *and learned new skills and knowledge that will prepare me to be a better medical doctor in the future*.

## Discussion

This section discusses the above findings in light of three key themes: congruence, professional-identity formation, and NPT training.

### Social, cognitive, and professional congruence

Our study found no significant differences between students’ perceptions of their near-peer tutors and their staff tutors, either in terms of facilitation or behaviour. These findings are consistent with those of a recent systematic review and meta-analysis of 16 studies of various health professions [26], which reported no meaningful difference in knowledge or skill scores among near-peer and expert teaching groups.

Also like ours, Khapre et al.’s [26] study found that students felt more comfortable with near-peer tutors because of the latter’s ability to create more relaxing learning environments, which in turn allowed them to ask and answer questions more openly and without fear of criticism. However, our participants also mentioned that near-peer tutors were better than staff ones at understanding their frustrations and seeing things from their perspective, which also made discussion easier.

Under the dimensional framework of peer teaching [1], our NPT programme can be described as *high-distance* (i.e., the tutors and tutees are one or more years apart), *large* (i.e., with three or more learners per group), and *high formality* (i.e., conducted in an academic setting and as part of a fixed curriculum). Guided by the psychological framework of NPT on both the cognitive and socio-psychological levels [1,27], our study confirms that both cognitive congruence and social congruence were important and valued factors in the NPT experience. Because of the relative similarity of near-peer tutors’ and tutees’ knowledge bases, and the former’s relatively recent experience of the same curriculum, tutors were able to sense the *zone of proximal development* proposed by Vygotsky [28] even better than faculty tutors could. And, from a socio-psychological perspective, our tutee participants felt that their near-peer tutors were able to provide the more open environment described above because of their similar social positions.

Prior studies have also suggested that cognitive and social congruence are not completely separate, but overlap [3]. While cognitive congruence reflects the similar knowledge bases that near-peer tutors and tutees share, it also involves role modelling and assimilation of the language and thought processes that experts use [29]. Similarly, while social congruence involves the similar social roles of near-peer tutors and tutees, it also appears to include the sharing of study strategies and learning experiences from outside the focal curriculum. This is consistent with the results of a quantitative factor-analysis study by Loda et al. [30], who revealed that a “non-judgmental learning atmosphere” and “informal communication” were associated with cognitive congruence, whereas “effectiveness” and “comprehensible explanations” belonged to social congruence (p. 4).

The above-mentioned overlap between cognitive and social congruence led Cianciolo et al. [3] to propose a broader construct, *professional congruence*, to represent the process of near-peer teachers supporting students’ “legitimate peripheral participation as future clinical learners” (p. 765). In our study, the 11 sampled near-peer tutors not only demonstrated their cognitive and social congruence with the students, but also shared their experiences as medical students with them, indicating that they “know more about the curriculum, know more about what they’re going to do, [and] know more about the style of learning and how to learn”.

In addition, the near-peer tutors brought up challenges that first-year medical students face when engaging in PBL, but which faculty tutors might miss; and the importance of addressing the difficulties the same students face when transitioning to more clinically relevant cases in their second semester. A few of the near-peer tutors mentioned that Year 1’s needed training in clinical reasoning, and in particular “how to ask patients for their histories, [and] how to come up with differential diagnoses”, as this would improve their preparedness for PBL discussions.

### Professional-identity formation

Literature on medical students’ professional-identity formation written from a social-learning perspective has proposed that they gradually evolve to “think, act, and feel like a physician” through various forms of socialisation, including social interactions in the learning environment, assimilating the behaviours of role models, and experiential learning [31,32]. NPT within the framework of a formal curriculum provided our senior students with opportunities for experiential learning [33], during which they played the role of a faculty member, took full responsibility for PBL tutoring and assessment, and assimilated how their own exemplar PBL tutors would engage in classroom facilitation [34]. Given the importance of teacher identity to future physicians’ multifaceted identities [35], NPT experience could thus facilitate this early process of professionalisation [36,37], and this benefit could be further magnified if students come to regard near-peer tutors as role models [38].

During this paper’s focal NPT experience, senior undergraduate medical students not only developed professional skills such as facilitating, monitoring, and assessing student learning, but also consolidated their own medical knowledge and skills. Together, these enhancements appear likely to facilitate their emerging identities as doctors within their medical-school community of practice [39]. In that community of practice [40], three domains were emphasised: *joint enterprise*, the process of working together toward a common goal; *mutual engagement*, interaction between individuals that gives rise to shared meanings; and *shared repertoire*, the common resources and vocabulary used to negotiate meaning and facilitate learning within a group [41].

It is also worth noting how socio-cultural aspects influenced students’ professional-identity formation, and how near-peer tutors and tutees viewed NPT [42]. In Asian educational settings, teachers are commonly treated as the only authority, and students tend to regard them as paragons [43,44]. This caused some initial challenges to our implementation of the focal programme. For example, one senior student who initially signed up to tutor for us dropped out after realising that the programme would be part of the formal PBL curriculum, citing serious concerns about ethics and academic integrity stemming from her own doubtful authority. Such comments could easily represent the concerns of an important subgroup of students or even faculty members about the legitimacy and legitimation of NPT. This idea tends to be confirmed by some opposition we initially received from the administrative and teaching team, concerning not only quality control of near-peer tutors’ teaching, as mentioned above, but also whether randomly assigning some students to NPT groups would unfairly disadvantage them. While we recognise that variations exist among faculty tutors with varied training backgrounds, levels and types of teaching experience, and teaching styles, it seems unlikely that as much variation would exist among senior undergraduate medical students. Nevertheless, it was specifically to address these concerns that we selected only one case from among a total of eight in the Cardiopulmonary and Renal Systems block for this pilot study of NPT.

The above-noted power dynamics also placed more stress on near-peer tutors to establish an image as a ‘perfect’ teacher. This echoes previous findings by Hundertmark and colleagues [45] that teacher-role demands are a key stressor for near-peer tutors. That prior study further suggested, however, that the more sessions near-peer tutors lead, the less psychological and physiological stress they will feel.

### Training and support for near-peer tutors

As noted earlier, a carefully designed peer-teaching certificate programme comprising four sessions taught by two trained teacher-educators was mandated for individuals wishing to perform NPT tutoring in PBL. Providing training for NPT has been shown to be important [46], but it cannot yet be described as well-developed. A systematic review revealed that, among 29 studies of NPT in undergraduate nursing education [27], only two mentioned the presence of training in pedagogical approaches [47]. The insufficiency of such training would undoubtedly pose additional challenges to near-peer tutors, including but not limited to lack of preparation and lack of confidence about taking on faculty-like responsibilities [48]. Thus, we concur that it is imperative to provide sufficient training for near-peer tutors, and embed it into curricula, not only in the content-knowledge area, but also in–at a minimum–pedagogy and facilitation skills.

## Limitations

Certain limitations of the current study should also be highlighted. First, it is based on one case study in one Asian medical school, with a unique curricular setting and socio-cultural background. The results therefore may not be generalisable to other medical schools or other socio-cultural settings. And second, the results were based on one relatively brief pilot study, rather than a long-term implementation of an NPT programme. Therefore, further longitudinal study of such programmes in medical schools should be conducted.

## Conclusions

The results of this exploration of a pilot integration of NPT into an undergraduate PBL medical curriculum suggest that, although students did not perceive their near-peer tutors’ skills or behaviours as differing from those of faculty tutors, near-peer tutees regarded their tutor groups as conveying the benefits of social and cognitive congruence to a greater degree than staff tutees did. These beneficial aspects of NPT were further confirmed from the near-peer tutors’ perspective. In addition, our data suggest that NPT experience could support near-peer tutors’ professional-identity formation by enhancing their understanding of teacher identity and strengthening the skills required of medical educators. Our findings also highlight the importance of adequate training to future near-pear tutors, especially in foundational pedagogical and facilitating skills. Future studies could usefully embed long-term NPT programmes in formal medical curricula and thereby provide richer evidence about NPT’s outcomes.

## Supporting information

S1 File Near-peer tutor interview protocol. (DOCX)
