# Supplementary material for: Near-peer teaching in problem-based learning: Perspectives from tutors and tutees
Source: PLoS One. 2022 Dec 14;17(12):e0278256. doi: 10.1371/journal.pone.0278256 (PMC9749983; doi:10.1371/journal.pone.0278256)
Supplement: S1 File — (DOCX) [file pone.0278256.s001.docx]

Near-peer tutor interview protocol

1. What motivated you to be an near-peer tutor?
2. How did your three tutorials go in general?
3. What did you gain most from the NPT experience?
4. What are the advantages and disadvantages of near-peer tutors as compared to staff tutors?
5. How did the NPT experience influence your learning or learning skills?
6. What do you think are important characteristics of a good PBL tutor?
7. What do you think of your identity shift from being a student to being a teacher?
8. What is your future career goal? Do you think teaching will be part of your career in the future?
9. How did the NPT experience influence your career goal?
10. What do you think of the peer teaching training provided? Any additional support you would like to receive?
